# Acoustic tracheal rupture provides insights into larval mosquito respiration

**DOI:** 10.1038/s41598-020-59321-8

**Published:** 2020-02-11

**Authors:** Herbert J. Nyberg, Kunihiro Muto

**Affiliations:** New Mountain Innovations, Inc., Old Lyme, CT 06371 USA

**Keywords:** Respiration, Entomology

## Abstract

Acoustic larviciding (AL) occurs by exposing mosquito larvae to acoustic energy that ruptures their dorsal tracheal trunks (DTTs) by the expulsion of gas bubbles into the body. In studying this technique, we serendipitously identified undescribed anatomical and physiological respiratory features. The classical theory of respiration is that the siphon and DTTs play obligate roles in respiration. Our results contradict the accepted theory that culicine larvae respire via atmospheric gas exchange. We identified an undescribed tracheal occlusion (TO) at the posterior extremities the DTTs. The TOs appear necessary for the acoustic rupture of DTTs; this constriction prevents the escape of energized gas from the siphon and allows the tracheal system to be pressurized. With a pressurized isolated tracheal system, metabolic gas exchange directly with the atmosphere is unlikely and could mostly occur through the chitin and setae. Future studies are needed to explore respiration and elucidate the mechanisms of oxygen absorption and carbon dioxide elimination.

## Introduction

Of all the disease-transmitting insects, mosquitoes are the greatest threat, as they transmit pathogens that cause diseases. In 2015, mosquitoes were responsible for 438,000 malaria-related deaths^1^. An outbreak of West Nile virus, in Queens, New York, in 1999 prompted the development of a new larvicide technique, acoustic larviciding (AL), in addition to traditional chemical larvicides^2^. While the exact mechanism of action of AL in mosquito larvae is unknown, tissue damage appears to be caused by vibrations that occur when the frequency of acoustic energy is matched to the resonant frequency^3^ of materials (i.e., water, air, tissue) inside the mosquito larvae, resulting in the vibration of these materials (similar to the shattering of crystal goblets by an opera singer). Exposing mosquito larvae to acoustic energy within a certain frequency band results in the rupture of the walls of the dorsal tracheal trunks (DTTs), causing the expulsion of gas into the body cavity, resulting in mortality, arrested larval development, or flightless adult mosquitoes. The DTTs are two significant tubes running the length of the abdomen acting as a central convergence of the tracheal system. AL, as a physical intervention, is effective against all larval stages with minimal lethality to off-target aquatic organisms^4,5^. It has been shown that treated larvae show pronounced damage to the DTTs^4^ suggesting that gas contained within these structures or the tracheal system is resonated by the acoustic vibration. However, the exact mechanism by which this phenomenon occurs is not clear within the framework of our current understanding of mosquito tracheal physiology.

In this study we attempted to elucidate the mechanism of action of AL using a precision research-grade, underwater acoustic transmitter. This device transmits controlled sonic energy to acoustically disrupt (i.e., rupture) the DTTs. This precise application of sound causes only the release of gas from the within the tracheal system into the body cavity while minimizing trauma to surrounding tissue. There is not enough power for this energized gas to rupture the exoskeleton and therefore remains trapped within the body cavity and can be readily measured for analysis. This technique allowed us to sever the tracheal trunks from the inside providing a novel perspective for evaluation.

This new approach revealed unexpected results shedding new light on the respiratory system. Observations from this technique impact the classical theory of mosquito larval respiration. This better understanding may potentially improve future efforts in controlling mosquito populations and the associated diseases they transmit.

## Results

### Source of released gas into the abdomen

**Figure 1:**
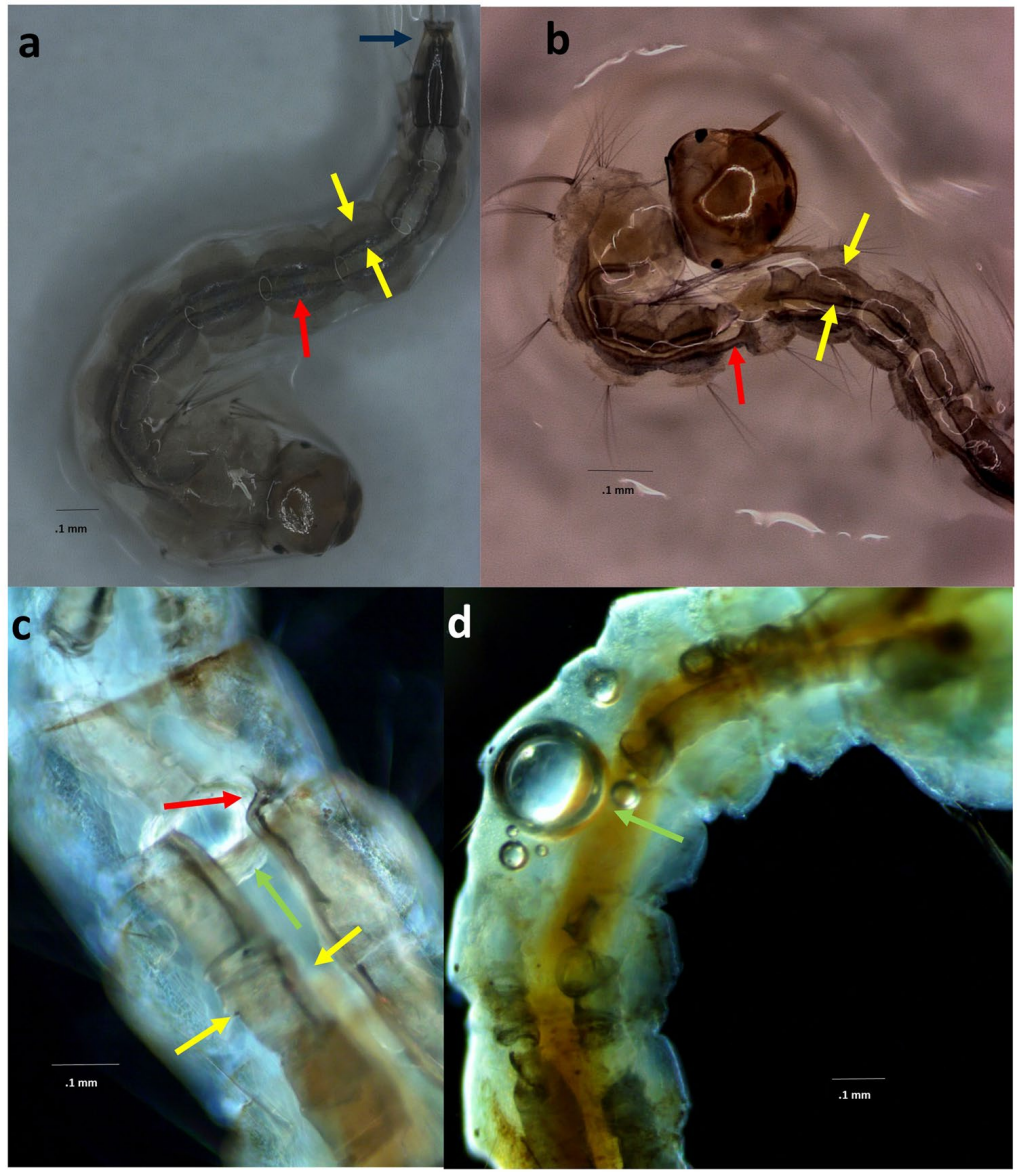
Examples of damage to DTTs as a result of AL. (**a**) Pre-exposure *Ae. aegypti* larva, active DTT (red arrow), future instar DTT (yellow arrows), Tracheal Occlusion (blue arrow). (**b**) AL exposed *Ae. aegypti* larva with pronounced trauma to both DTTs. Note that the internal active DTT (red arrow) appears to be the source of the expelled gas within the larger fluid-filled DTTs (yellow arrow) of the future instar. (**c**,**d**) Culex pipiens larva showing destruction of the DTTs. Liberated gas bubble (green arrow) are visible within the abdomen.

We found that AL treatment causes instantaneous and irreversible trauma to mosquito larvae. Before and after acoustic exposure of *Aedes aegypti* and *Culex pipiens* larvae depicting the impact on internal structures such as the active and future instar DTTs are shown in Fig. 1. Unexposed specimens’ (Fig. 1a) DTTs are intact with the active DTTs internal to the liquid filled future instar DTTs. Observations of acoustically treated larvae (Fig. 1b,c) revealed trauma to the DTTs with outward flares of the active DTTs. The future instar DTTs sustained damage and gas bubbles were observed within the abdomen. We observed this breach to be typically located at the interface of the abdominal segment (the same point where the DTTs separates into segments during molting).

### The tracheal system is maintained at an elevated pressure

**Figure 2:**
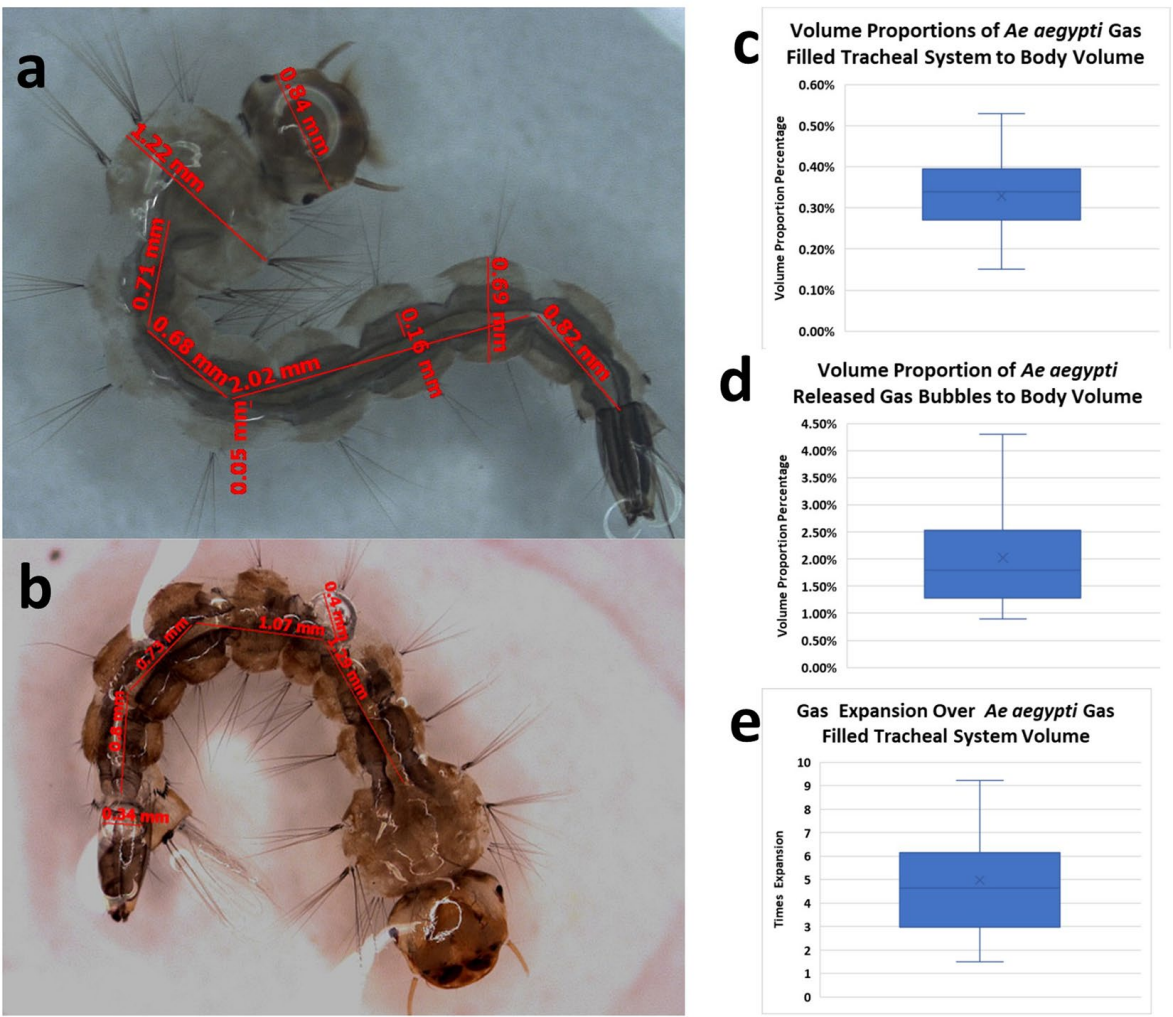
Analysis of pressure in the tracheal system of *Ae. aegypti* mosquito larvae. (**a**) Pre-exposed measurements of the larvae body and active DTT (millimeter dimensions in red) diameter and lengths. (**b**) Post-exposure measurements (millimeter lengths in red) of the length and diameter of the active DTT and the diameters of the expanded gas bubbles. (**c**) Volume proportions of gas filled tracheal system to body volume. (**d**) Volume proportion of released gas bubbles to body volume. (**e**) Gas expansion over gas filled tracheal system. n = 37.

The gas volume expelled from the previous test appeared relatively large compared to the volumes of the DTTs, indicating the possibility that the tracheal system was maintained at a higher pressure than the abdomen. Measurements and analysis of physical features and visible gas volumes before and after acoustic treatments of *Ae aegypti* larvae were conducted (Fig. 2a,b). Exposures and measurements were conducted on thirty-seven (n = 37) specimens see Supplemental Data Files. The percentage of *Ae aegypti* gas filled tracheal system to body volume (Fig. 2c) had a mean of 0.33%. The percentage volume of *Ae aegypti* released gas bubbles to body volume (Fig. 2d) had a mean of 2.02%. The gas expansion over *Ae aegypti* gas filled tracheal system (Fig. 2e) revealed a mean expansion of 4.97 times.

### Morphological evaluation identified a previously unreported Tracheal Occlusion

We endeavored to explain how the tracheal system presented an elevated pressure, as the traditional understanding of mosquito larval tracheal systems suggests that the tracheal system is open to the environment via the siphon.

**Figure 3:**
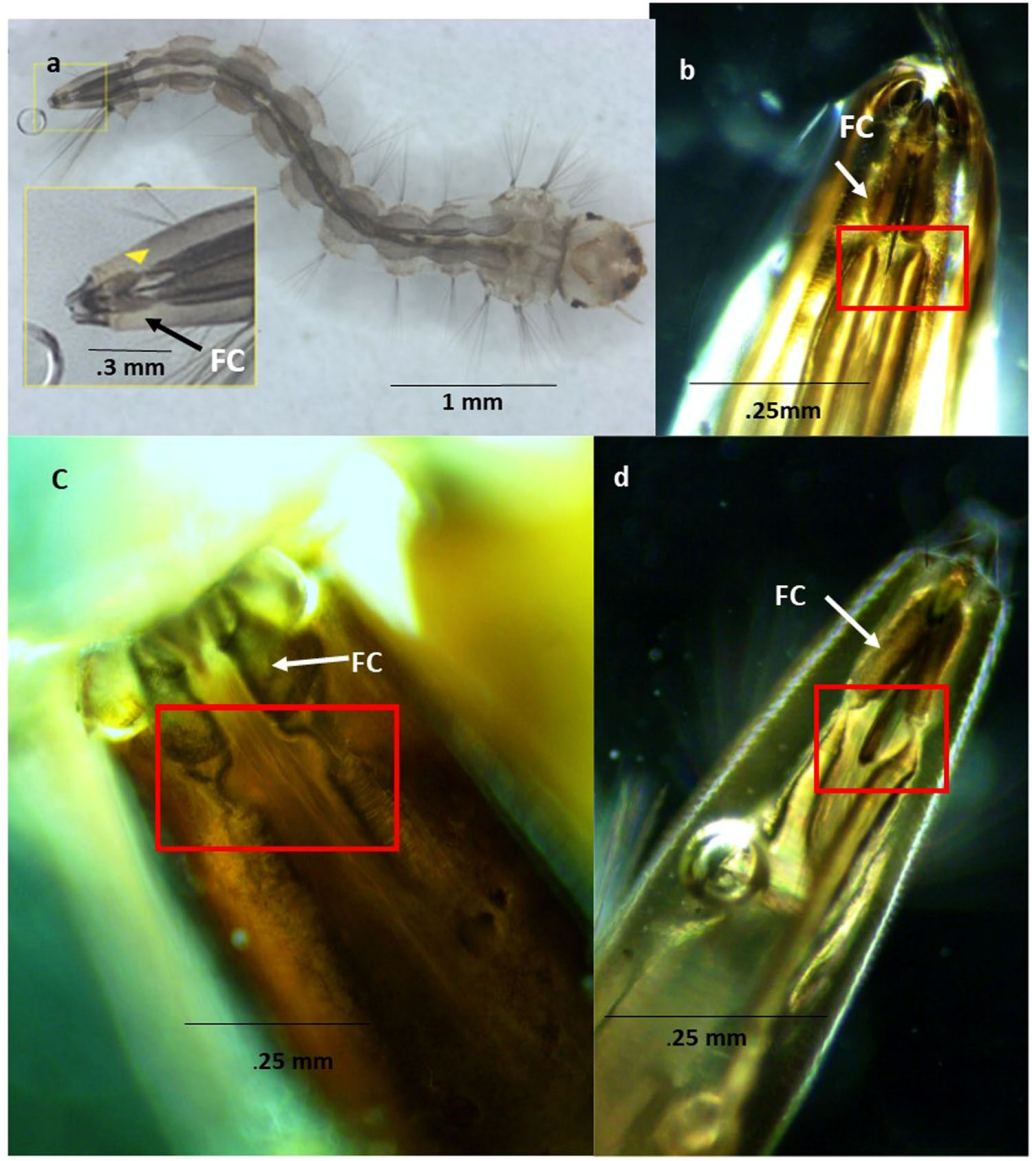
Distinctive tracheal occlusions (TOs) of the DTTs are present in the siphon. Unreported constriction at the posterior end of the DTTs are identified. (**a**) *Ae. aegypti* (with siphon enlarged in the inset image) yellow triangle points out the TO. (**b**) Siphon of *C. pipiens*. The TO is located within the red box. (**c**) Siphon of *Toxorhynchites* spp. siphon. (**d**) *C. pipiens* following exposure to the AL treatment. This image shows the gas bubble liberated from the DTTs, suggesting that the occlusion is strong enough to prevent gas from exiting to the environment, and may be stronger than the DTTs themselves. Felt chamber labeled FC.

**Figure 4:**
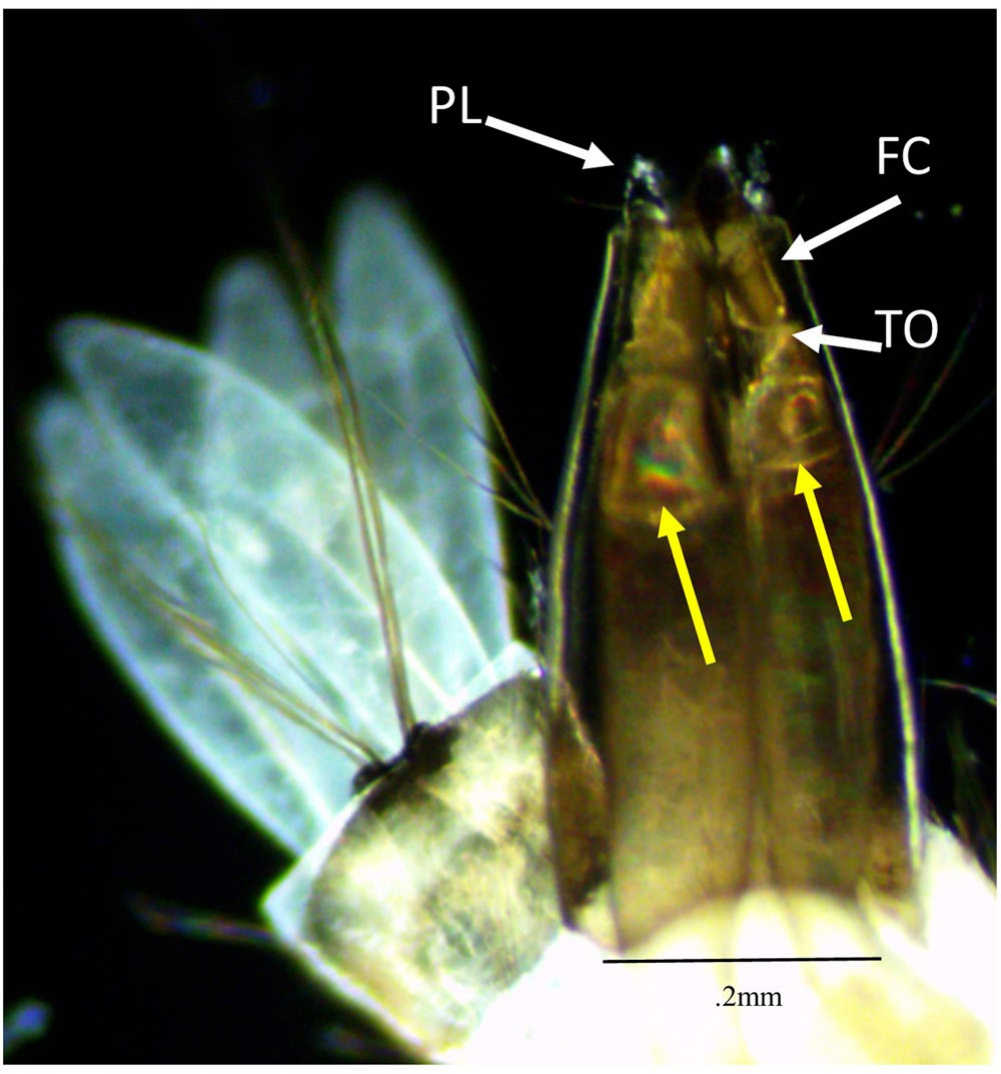
Tracheal gases blocked from passing through the Tracheal Occlusion (TO). Note the gas (yellow arrow) internal to the DTTs is differentiated from liquid by higher transparency and refraction of light showing spectrum colors. This indicates the TO restricts the movement of gas between the tracheal system and external environment. The PLs are in a partial open condition. *Ae. aegypti* perispircular lobes (PL), Felt Chamber (FC).

Our investigation revealed a previously undescribed Tracheal Occlusion (TO) located between the posterior end of the DTTs and the Felt Chambers (FC) that was evident under a light microscope at 4x magnification in third and fourth instar culicine larvae (Fig. 3). The persistence of the TO in maintaining its integrity through the treatment beyond the rupture of the DTTs walls indicates it is strong. Presence of the structure (Fig. 3d) taken after treatment is supporting evidence. We also observed that after exposure, the gas remaining in the DTTs did not pass beyond the TO even when the perispicular lobes (PL) were partially open (Fig. 4).

We observed that this constriction did not change its circumferential dimensions when the mosquito was moving or with the opening or closing of the PLs (see Supplemental Video). We observed the DTTs lateral movements including folding into the FC but no opening of the occlusion. The DTTs chitinous construction continued to the tip of the occlusion with decreasing taenidia diameters to the point of occlusion (Fig. 3b). In all thirty-seven specimens observed the TO totally restricted the release of hemolymph, tissue or gas from the siphon to the exterior. In several specimens (larvae, 5, 22, 24, 26, 27, 28, 32), gas bubbles are found in the siphon but were stopped from movement beyond the TO (Fig. 4, Supplement Data File).

### A severed dorsal tracheal trunk at the Tracheal Occlusion-Felt Chamber transition demonstrates the importance of the tracheal occlusion for acoustic larviciding

We severed the siphons of five larvae at the TO-FC interface and exposed these larvae to acoustic energy. The five control specimens presented ruptured DTTs. The five severed specimens did not result in resonance-induced trauma, as no tissue damage or visible gas bubbles were observed in the hemocoel. We did observe slight movement when acoustic energy was applied but the not typical reaction from the control group. A video of a live specimen after exposure that showed no damage to the DTTs or gas bubbles in the abdomen is in the Supplemental Material.

### Survival with an impaired, severed or isolated siphon is possible

**Figure 5:**
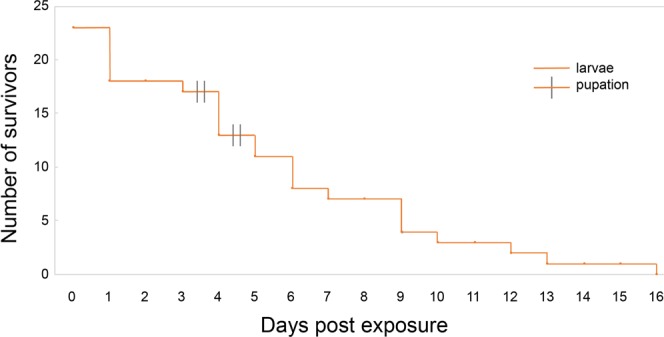
Survival of *Ae. aegypti* larvae (plotted using Kaplan Meier survival curve) (n = 23) with ruptured DTTs after exposure to sublethal acoustic energy. Some mosquitoes died within 24 hours of acoustic exposure, others survived for an extended period, with visible damage to their DTTs. Note, four treated mosquitoes pupated; these mosquitoes were removed from further observation and are shown on the graph as pupation events.

As outlines for the previous experiments, the delivery of a sublethal dose of acoustic energy ruptured the DTTs while minimizing trauma to other tissues (i.e., the gut and exoskeleton). Mosquitoes exposed to this technique did not die instantly, and many of them survived for long periods of time despite severed DTTs. In our experiments, 31 4^th^-instar *C. pipiens* were exposed to acoustic energy, and DTT damage was visually confirmed in each mosquito. Despite this damage, 11 of the larvae survived to pupation, which occurred 2–4 days after treatment. In a replicate experiment, 44, 4^th^-instar *C. pipiens* larvae were treated, and 13 larvae pupated 2-8 d after exposure. Furthermore, DDT-damaged larvae exhibited the same behaviors as the control mosquitoes (i.e., swimming, feeding, and positioning in the water column). When these experiments were repeated on 23 third-instar *Ae. aegypti* larvae, 18 survived for 24 hours, and four larvae pupated. The remaining 14 larvae displayed arrested development with the longest-lived larva survived for 15 days with damaged DTTs (Fig. 5).

During additional sublethal exposure experiments, a few individuals who exhibited arrested larval development lived for exceptionally long periods of time (despite compromised DTTs). Supplement 2a shows a microscopic examination of *C*. *pipiens* larvae with pronounced damage to the DTT that survived for over 20 days in a cool environment with no supplemental nourishment.

Obstructing the siphon by applying paraffin to the PLs of *C*. *pipiens* and *Ochlerotatus cantator* (Supplement Fig. 2b) still permitted survival in very shallow water. For larvae maintained in shallow water (<2.4 mm), this treatment had no observable impact on larval activity, other than the inability of the larvae to attach themselves to the surface of the water due to the paraffin. We observed bottom-feeding mosquito larvae assume a slight inclined angle when they feed. In this shallow water, their natural inclination maintained the siphon and ventral fan near the surface. Larvae maintained in water that was five cm deep were observed to be unsuccessful in attempts to attach to the water surface before moving to the bottom of the water column. *C. pipiens* exhibited decreased activity after 48 hours and death after 72 hours. The 12 control and treated *Oc. cantator* specimens survived over five days. The deep-water control group exhibited normal bottom-feeding and surface-resting behaviors at expected intervals. In shallow water, the control mosquitoes constantly remained suspended from the surface while scavenging the bottom for food. Although the shallow-water treatment group did not suspend themselves from the surface, their normal inclination positioned the blocked siphon and ventral fan adjacent to the surface.

## Discussion

Mosquitoes transmit a diverse range of pathogens^6–10^, and thus, an improved understanding of mosquito physiology and the development of novel control strategies are critically important. AL has afforded us new insights into mosquito larval respiration. The application of AL resulted in the expulsion of gas bubbles that originated from within the tracheal system. When the impinging acoustic frequency is equal to the resonant frequency of the gas within the tracheal system, that gas maximally absorbs acoustic energy and begins to pulsate in synchrony with the impinging frequency. As energy continues to be applied, the amplitude of the pulsation increases to the point of rupturing the DTTs. By adjusting the amplitude and pulse length of our acoustic signal, we observed the earliest manifestations resulting from AL, the severing of the DTTs, with minimal collateral tissue trauma. This novel technique allowed us to reveal several new aspects of the larval tracheal system. Our study has five major outcomes. First, we improved our understanding of the mosquito tracheal system, including the possible isolation of the tracheal system from the atmosphere. Second, we presented a potential mechanism for the maintenance of pressure during impingement, which damages the DTTs (as opposed to gas venting through the siphon). Third, we provided new insights into the morphology of the siphon (the identification of the TO). Fourth, we confirmed that the damage-inducing mechanism of action of AL is the acoustic resonance of the gas within the tracheal system. Finally, we observed that the siphon does not play an obligate role in respiration for the following reasons: The TO appears to isolate and maintain the tracheal system at an elevated pressure thus making it at best an inefficient port for the two-way exchange of metabolic gasses. Larvae with completely blocked spiracles or severed DTTs continued to live for long periods of time. After acoustic exposure we did not observe any hemolymph (liquid, solids or gasses) pass by the TO as would have been expected if the siphon was open to the atmosphere.

Margaret L. Keister reported “… a survey of the literature (see Wigglesworth,’31) shows that there are numerous gaps and contradictions in our knowledge of insect tracheal systems”^11^. Our anatomical findings complement a recent revival of interest in mosquito respiratory physiology. However, some conflicts still exist^12–16^. Accordingly, it is important to define some terms used herein. The “FC” is identified by Keilin, Tate and Vincent as the terminal chamber between the spiracles and DTT^17^. An appreciation of the physiology of the DTTs is important in analyzing gas volumes. Regarding “active DTTs”, during the development of a given instar between molts, the DTTs are comprised of a chitinous partially gas-filled trunk; the active DTTs are enclosed in the large and fluid-filled living-tissue trunks of the future-instar DTTs (Supplemental 1a). However, the composition of this fluid is unknown^18,19^ and needs further investigation. The active DTTs are withdrawn during molting.

While resting on the water surface with the five PLs extended, it appeared that mosquito larvae were in an ideal position to freely exchange metabolic gases, intake oxygen and expel carbon dioxide. Our results indicated that there was not an obligate need for this, and beyond the incidental cuticular exchange of gas with the atmosphere in the atrium, the direct exchange of tracheal gases with the atmosphere (breathing) is unlikely.

By comparing various anatomical and physical characteristics before and after acoustic exposure we identified the tracheal system to be at an elevated pressure. Mosquito larvae are nearly neutrally buoyant^20^. They are composed of solids, liquids and gasses. In order to maintain their buoyant condition, the volume proportions of the gas filled tracheal system to body volume must fall within a precise range. As reported by Ha, this percentage for *Anopheles sinensis* larvae was.34%^16^. The results of our observations and calculations using 37 *A. aegypti* samples was.33%. This is expected as most of the body is liquid therefore only a small percentage of body volume could be gas. We calculated that the mean post acoustic exposure proportion of gas bubbles to body volume is 2.02%. This represents and expansion of 5.9 times meaning the initial pressure in the tracheal system was high. The mean direct expansion of the gas bubble was 5.0 times that of as original tracheal size. The function of pressurization in the DTT is unknown, and its potential relationship with tracheal filling or emergence should be further studied.

A pressurized tracheal system makes the inhalation of oxygen difficult if not impossible. The TO is quite strong because it involves acoustically induced pressure oscillations that exceed the ability of the DTTs to contain them. The dimensions of the TO between surface-resting or submerged larvae do not change, suggesting that the restriction prohibits the exhaust of carbon dioxide. Dissection to sever the siphon anterior to the FCs also rendered AL ineffective, indicating that the TO is a necessary structure for the success of AL.

The condition of total dependence on cuticular (and/or gill- or filament-supported) respiration in immature aquatic insects is common and present in many close relatives of mosquitoes. Culicinae are joined by seven other families in the infraorder Culicomorpha whose immatures all respire in total submergence^21^. The consideration that the siphon plays only a vestigial role in respiration is not without precedent. *Corethra* (also called midge and of the family *Chaoboridae*) were classified as mosquitoes until the early 1960s^22^; today, they are considered taxonomically separate but are thought to share a common ancestor^21^. *Corethra* and mosquito larvae share common physiological traits, and many species look very much alike, including the presence of an apparent larval siphon^21–26^. Krogh found that larvae of the genus *Corethra* appeared to respire through only the skin and concluded that this organism fills its air sacks with gas from a non-atmospheric source (i.e., tissues)^27^. He also noted that the DTTs did not contain air and that the connected bladders appeared to have no respiratory function. *Mochlonyx* spp. (also in *Chaoboridae*) possess a siphon (Supplemental 3) but do not come to the surface to breathe^25,26,28,29^; hence, the siphon is clearly not used for the exchange of atmospheric gases in this species. As noted by Förster and Woods, and Keister and Buck, filling of the tracheal system with gasses from an endogenous source has been observed in a variety of organisms^11,18,19^.

It has been previously reported that mosquito larvae can survive for long periods of time without access to the atmosphere, indicating that aquatic respiration is possible^30–36^. For example, Macfie (1917) demonstrated that submerged larvae (isolated from surface air) of certain mosquito species can live for 20 days if the aquatic medium is adequately aerated. Mosquito larvae are found in aquatic environments with variable levels of dissolved oxygen^32,37–40^ and can survive in water with low levels of dissolved oxygen concentrations (e.g., 0.04 to 1.63 mg/L). However, we propose that previous reports on mosquito survivability in low dissolved oxygen concentrations failed to consider that the dissolved oxygen content at the surface of water is higher from that further down in the water column. Vacha and others noted that the concentration of dissolved oxygen at the air-water interface was enhanced^41,42^.

Mosquito larvae in a surface-resting posture position their body, especially their ventral fan, in the stratum with the highest oxygen concentration while simultaneously conserving energy. This may be an important behavior in sourcing metabolic oxygen. Therefore, future investigations should focus on the role of the ventral fan in mosquito larval respiration. Our observations may question the commonly accepted mode of action (suffocation) of petroleum surfactants. According to the literature, the most rapid mode of action may be neurological disruption, not suffocation^30^. Suffocation normally takes a long time, which may be related to reduced surface oxygen concentrations or the direct impairment of cutaneous gas exchange^43–49^.

Our results show that the tracheal system is isolated and maintained at an elevated pressure, thus making the free exchange of metabolic gases with the environment unlikely. We report a previously undescribed TO that appeared to isolate the tracheal system and enabled acoustic energy to intensify and rupture the DTT. Our findings are not without precedence; as with other family members in the infraorder Culicomorpha, it is common for immatures to totally source metabolic oxygen from and expel waste gases directly into the water.

These findings appear to contradict the fundamental understanding of culicine larval respiration. Additional tests and research evaluating gas movements through the environment as well as within the animals needs to be done. With the new physical and physiological information from this study, possibly novel methods to control this deadly insect will be developed.

## Materials and Methods

### “Mosquitoes” and “sample preparation”

Because of the fragile nature of the gas within larval mosquitoes, all the samples reported here were not subject to any artificial preparations, such as stains, or fixed with any gels or semisolids. All the specimens were either alive or had been recently exposed to acoustic energy. To accurately observe the dimensions, cover slides were not used to constrain the animals while the images were captured.

### Precision acoustic ablation of DTTs for gas analysis

To determine the source of the gas, characterize tracheal pressurization and observe longevity after exposure, a New Mountain SD-Mini acoustic amplifier was used to generate an acoustic signal for transmission in the water. To produce sound in the water, two ring piezoelectric transducers (under a water loudspeaker) (five-cm diameter) resonate at 24 kHz was used. To accommodate the full frequency spectrum of 18 to 30 kHz, the transducer was electrically matched with a transformer and inductors.

To determine the source of the gas and volume measurements, *Ae. aegypti* larvae were simultaneously hatched and reared at the Connecticut Agricultural Experiment Station; a group of 50 specimens of approximately equal size and length were selected from the hatched larvae. The volumes of the active DTTs prior to exposure to acoustic energy were determined by measuring the tracheal diameter at six points to determine the mean active DTT diameter in five specimens of identical size. The mean active DTT diameters for 2^nd^ instar larvae were 0.03 mm and 3^rd^- 4^th^ instar larvae were 0.04 mm. This method of determining the pre-exposure volume is accurate when combined with the post-exposure length, as length is not compromised during acoustic exposure since the rupture is always circumferential. Additionally, the postexposure measurements of the active DTT diameter did not differ from the pre-exposure measurements. The overall gas filled DTT length was measured in each individual, and the volume was calculated using the mean preexposure diameter of the DTTs.

An acoustic signal pulse length of 3 ms (23,000 cycles/sec × 0.003 s = 46 cycles per pulse) and an amplifier output of 35% were used for 3^rd^- to 4^th^-instars, while a pulse length of 2 ms (25,500 cycles/sec × 0.002 s = 51 cycles per pulse) was used for 2^nd^-instar larvae. Larvae were placed 15 cm away and in line with the midpoint of the transducer. The precision acoustic source was immediately activated for one pulse length, and the larvae were immediately removed from the tank with a pipette and placed on a glass slide. Measurements were made using a Zeiss Discovery V12 Stereo Microscope with an Axiocam USB camera and associated AxioVision software V4.8 (Zeiss, Oberkochen, Germany) was used. The volume of the dorsal tracheal trunk was calculated as $\Pi \,\ast \,{{\rm{radius}}}^{2}\,\ast \,{\rm{length}}$, with the assumption that the DTT was cylindrical. The volume of expanded gas was calculated as $\frac{3}{4}\,\ast \,\Pi \,\ast \,{{\rm{radius}}}^{3}$. When there was more than one bubble, the volumes of all the bubbles were summed. Body displacement was calculated by measuring the diameter of the head and thorax, the length and diameter of the abdomen and siphon.

For all other anatomical observations, an OMAX 40 × -2000X compound trinocular biological microscope with a dark-field light source was used. Images (video and still) were captured with a three-megapixel USB camera, and Toupview software on a Windows 10 operating system.

### Damming the siphon with paraffin

Third instar wild *C. pipiens* and *Oc. cantator* were collected locally in Old Lyme, Connecticut, from 55-gallon drums. The specimens were covered and constrained under a microscope slide to expose only the siphon. A drop of melted paraffin was adhered to the PL (Supplemental Image 2b). Care was taken not to cover any portion of the ventral brush (fan), anus, or any other setae. Four beakers were filled with water collected from the mosquitoes’ natural habitats (two beakers filled to a depth of 2.4 mm and two filled to a depth of five cm). For *C. pipens* two replicates of three larvae and for *Oc. cantator* two replicates of four larvae were placed in each beaker and observed for activity or mortality every six hours. Mortality was determined using World Health Organization, “Guidelines for laboratory and field testing of mosquito larvicides”^50^.

### Severing the siphon at the TO FC interface

*C. pipiens* larvae from the same source were observed and dissected using 10x magnification and a surgical scalpel. The siphons of three 3^rd^-instar larvae was cut anterior to the TO. The acoustic protocol exposed the specimens to the full spectrum of frequencies, ranging from 18 kHz to 30 kHz. The frequencies were applied in 500 Hz increments, with a pulse length of 400 cycles.

### Post exposure longevity evaluation

At the University of Notre Dame, South Bend, Indiana, *C*. *pipiens* and *Ae. aegypti* were obtained from laboratory colonies and exposed to acoustic energy as described above. Individual post exposure larvae were removed with a pipette and examined for tracheal damage with a microscope. Damaged, living specimens were transferred to a water filled rearing tray for observation. The numbers of individuals of both species exhibiting immediate mortality (determined by WHO WHOPES guidelines^50^) were noted. Specimens were observed every 12 hours for activity, molting and mortality.

## Supplementary information

Supplementary information

Dataset 1.

Tracheal Occlusion (TO)lateral motion during siphon movement.

Severed Siphon specimin post acoustic exposure.

## Data Availability

All data is available without restrictions.

## References

[1] World Health Organization. Mosquito-borne diseases, https://www.who.int/neglected_diseases/vector_ecology/mosquito-borne-diseases/en/ (2019).

[2] Nyberg, M. H. & Nyberg H. J. Method for killing mosquito larvae*. U.S. Patent* 6298011 (2001).

[3] Urick, R. J. *Principles of Underwater Sound*. McGraw-Hill, New York (1975).

[4] Fredregill CL, Motl GC, Dennett JA, Bueno R, Debboun M (2015). Efficacy of two larvasonic units against culex larvae and effects on common aquatic nontarget organisms in Harris County, Texas. Journal of the American Mosquito Control Association.

[5] Britch SC, Nyberg H, Aldrich RL, Swan T, Linthicum KJ (2016). Acoustic control of mosquito larvae in artificial water containers. Journal of the American Mosquito Control Association.

[6] Andreadis, T. G., Thomas, M. C. & Shepard, J. J. *Identification Guide to the Mosquitoes of Connecticut*. The Connecticut Agricultural Experiment Station, New Haven (2005).

[7] Turell MJ (2005). An update on the potential of North American mosquitoes (Diptera:Culicidae) to transmit West Nile Virus). Journal of Medical Entomology.

[8] Watts KJ, Courtney CH, Reddy GR (1999). Development of a PCR- and probe-based test for the sensitive and specific detection of the Dog Heartworm,Dirofilaria immitis, in its mosquito intermediate host. Molecular and Cellular Probes.

[9] Dimopoulos G (2003). Insect immunity and its implication in mosquito–malaria interactions. Cellular Microbiology.

[10] Armstrong PM, Andreadis TG (2010). Eastern equine encephalitis virus in mosquitoes and their role as bridge vectors. Emerging Infectious Diseases.

[11] Keister ML (1948). The morphogenesis of the tracheal system of sciara. Journal of Morphology.

[12] Lee SJ, Kim JH, Lee SC (2018). Effects of oil-film layer and surfactant of the siphonal respiration and survivorship in the fourth instar larvae of Aedes togoi mosquito in laboratory conditions. Scientific Reports.

[13] Lee S, Kim JH, Lee SJ (2017). Floating of the lobes of mosquito (Aedes togoi) larva for respiration. Scientific Reports.

[14] Ha YR, Yeom E, Ryu J, Lee SJ (2017). Three-dimensional structures of the tracheal systems of Anopheles sinensis and Aedes togoi pupae. Scientific Reports.

[15] League GP, Hillyer J (2016). Functional integration of the circulatory, immune, and respiratory systems in mosquito larvae: Pathogen killing in the hemocyte-rich tracheal tufts. BMC Biology.

[16] Ha YR, Ryu J, Yeom E, Lee SJ (2017). Comparison of the tracheal systems of Anopheles sinensis and Aedes togoi larvae using sychrotron X-ray microscopic computed tomography. Microscope Research and Technique.

[17] Christophers, Sir S. R. *Aedes Aegypti (L.) The Yellow Fever Mosquito*. The University Press, Cambridge, 221 (1960).

[18] Keister ML, Buck JB (1949). Tracheal filling in sciara larvae. The Biological Bulletin.

[19] Forster TD, Woods HA (2013). Mechanisms of tracheal filling in insects. Biological Reviews.

[20] Tuno N (2004). Diving ability of Anopheles gambia (Diptera: Culicidae) lavae. Journal of Medical Entomology.

[21] Merit, R. W. & Cummins, K. W. *An Introduction to the Aquatic Insects of North America*. Kendell/Hunt Publishing Company, New York (1996).

[22] Borkent CJ, Borkent A (2008). Description and phylogenetic interpretation of chromatophore migration from larval air sacs to adult structures in some Chaoboridae (Diptera). The Canadian Entomologist.

[23] Borkent A (1979). Systematics and bionomics of the species of the Subgeneus Schdonophasma Dyar and Shannon (Chaoborus, Chaoboridae, Diptera*)*. Quaestiones Entomologicae.

[24] Tolloch GS (1939). A Key to the mosquitoes of Massachusetts. PSYCHE.

[25] Lake RW (1969). Life history habitat and taxonomic characters of the larva of mochlonyx fulginosus. Entomological News.

[26] Norbert, B. *Mosquitoes and Their Control*. Springer-Verlag, Berlin, Heidelberg, (2010).

[27] Krogh A (1911). On the hydrostatic mechanism of the Corethra larva with an account of methods of microscopical gas analysis. Acta Physiologica.

[28] James HG (1957). Mochlonyx velutinus (Ruthe) (Diptera: Culicidae), an occasional predator of mosquito larvae. The Canadian Entomologist.

[29] Wojtal-Frankiewicz A, Frankiewicz P, Jurczak T, Grennan J, McCarthy TK (2010). Comparison of fish and phantom midge influence on cladocerans diel vertical migration in a dual basin lake. Aquatic Ecology.

[30] Richards AG (1941). Differentiation between toxic and suffocating effects of petroleum oils on larvae of the house mosquito. Transactions of the American Entomological Society.

[31] Reiter P (1978). The influence of dissolved oxygen content on the survival of submerged mosquito larvae. Mosquito News.

[32] Macfie JWS (1917). The limitations of kerosene as a larvicide, with some observations on the cutaneous respiration of mosquito larvae. Bulletin of Entomological Research.

[33] Lee SJ, Kim JH, Lee SC (2018). Effects of oil-film layer and surfactant of the siphonal respiration and survivorship in the fourth instar larvae of Aedes togoi mosquito in laboratory conditions. Scientific Reports.

[34] Wigglesworth VB (1938). The absorption of fluid from the tracheal system of mosquito larvae at hatching and molting. Journal of Experimental Biology.

[35] Wigglesworth VB (1933). The function of the anal gills of the mosquito larva. Journal of Experimental Biology.

[36] Fox HM (1921). Methods of studying the respiration exchange in small aquatic organisms, with particular reference to the use of flagellates as an indicator for oxygen consumption. The Journal of General Physiology.

[37] Low VL (2012). Nationwide distribution of culex mosquitoes and associated habit characteristics at residential areas in Malaysia. Journal of the American Mosquito Control Association.

[38] Oyewole IO (2009). Physico-chemical characteristics of Anopheles breeding sites: Impact on fecundity and progeny development. African Journal of Environmental Science and Technology.

[39] Thullen JS, Sartoris JJ, Walton WE (2002). Effects of vegetation management in constructed wetland treatment cells on water quality and mosquito production. Ecological Engineering.

[40] Silberbush A, Abramsky Z, Tsurim I (2015). Dissolved oxygen levels affect the survival and developmental period of the mosquito Culex pipiens. Journal of Vector Ecology.

[41] Vacha R, Slavicek P, Mucha M, Finlayson-Pitts BJ, Jungwirth P (2004). Adsorption of atmospherically relevant gases at the air/water interface:  free energy profiles of aqueous solvation of N_2_, O_2_, O_3_, OH, H_2_O, HO_2_, and H_2_O_2_. Journal of Physical Chemistry.

[42] Matear, R. J., Hirst, A. C. & McNeil, B. I. Changes in dissolved oxygen in the Southern Ocean with climate change. *Geochemistry, Geophysics, Geosystems* **1** (2000).

[43] Bacaër Nicolas (2011). Ross and malaria (1911). A Short History of Mathematical Population Dynamics.

[44] McMullen AI, Reiter P, Phillips MC (1977). Mode of action of insoluble monolayers on mosquito pupal respiration. Nature.

[45] Murray DRP (1936). Mineral oils as mosquito larvicides. Bulletin of Entomological Research.

[46] Corbet SA (2000). Surface films as mosquito larvicides; Partitioning the mode of action. Entomologia Experimentalis et Applicata.

[47] Watson GI (1941). A physiological study of mosquito larvae which were treated with anti-malarial oils. Bulletin of Entomological Research.

[48] Hacker, H. P. How oil kills anopheline larvae. *F. M. S. Malaria Bureau Reports* **3** (1925).

[49] Micks DW, Rougeau D (1976). Entry and movement of petroleum derivatives in the tracheal system of mosquito larvae. Mosquito News.

[50] World Health Organization. *Guidelines for laboratory and field testing of mosquito larvicides* (2005).

