# Supplementary material for: Acoustic tracheal rupture provides insights into larval mosquito respiration
Source: Sci Rep. 2020 Feb 11;10:2378. doi: 10.1038/s41598-020-59321-8 (PMC7012908; doi:10.1038/s41598-020-59321-8)
Supplement: Supplementary file 1 — Supplementary information [file 41598_2020_59321_MOESM1_ESM.pdf]

Acoustic tracheal rupture provides insights into larval mosquito respiration (Supplemental)

*Herbert J. Nyberg (sales@newmountain.com)
New Mountain Innovations, Inc.
Old Lyme, CT 06371
Kunihiro Muto (kuni.muto@gmail.com)
New Mountain Innovations, Inc.
Old Lyme, CT 06371

* Corresponding author

Supplemental

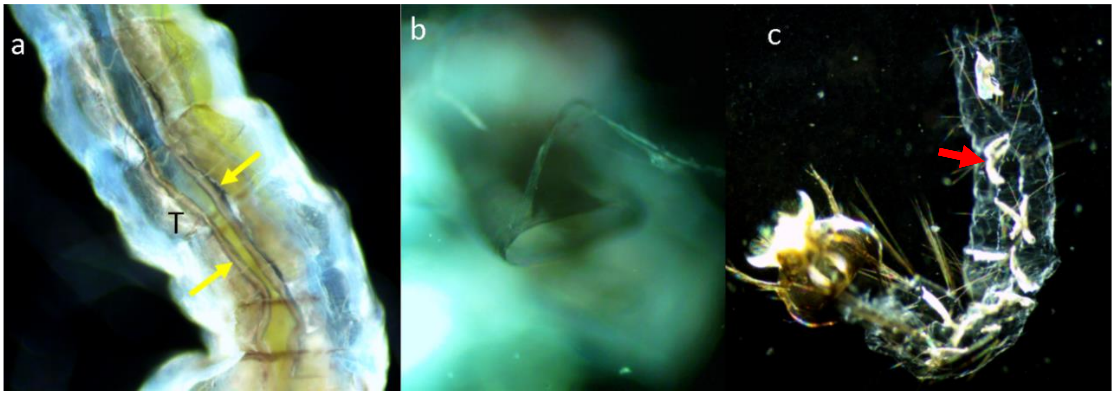

Supplemental 1. Dynamic changes in mosquito larval physiology. a. *C. pipiens* larvae, liquid filled future DTT labeled, T and active-DTT with arrow. b. Sectioned 4th instar *Toxorhynchites* spp. abdomen at 5th segment showing active-DTT chitin construction and some of the individual rings or taenidia separated during the incision. c. Cast skin following a molt of a 4th instar *C. pipiens* larvae, note the 16 segments (red arrow) of the active DTTs are withdrawn at molting.

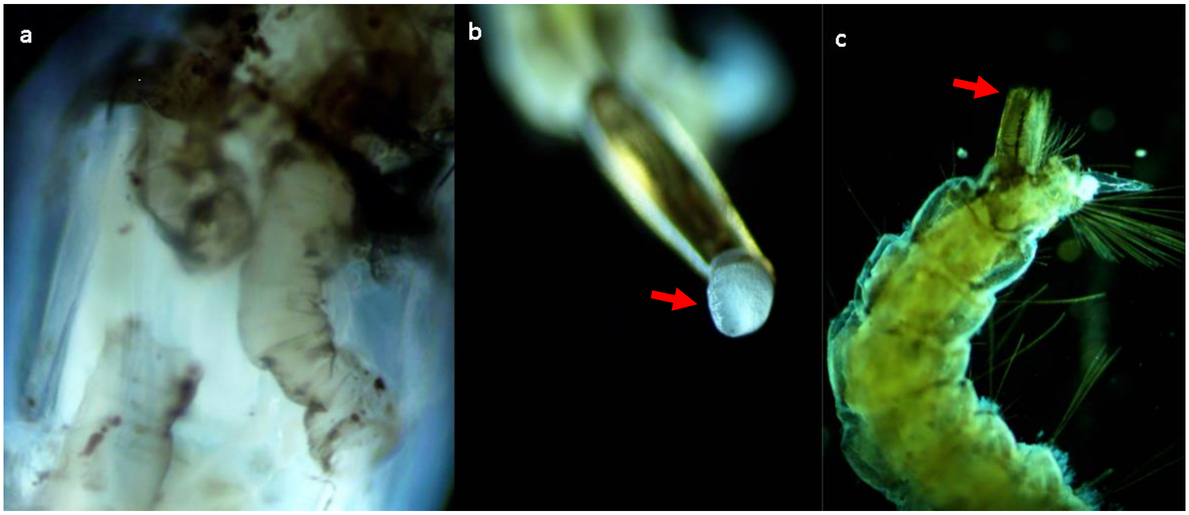

Supplemental 2. *C. pipiens* larvae were treated with precision (sublethal) acoustic larvicide, paraffin damming, and siphon severed a. *C. pipiens* image from living larvae 21 days after acoustic treatment. b. Perispiracular lobes of *C. pipiens* dammed with paraffin (red arrow) to isolate the siphon from the atmosphere. c. *C. pipiens* severed siphon (red arrow) anterior to the felt chamber.

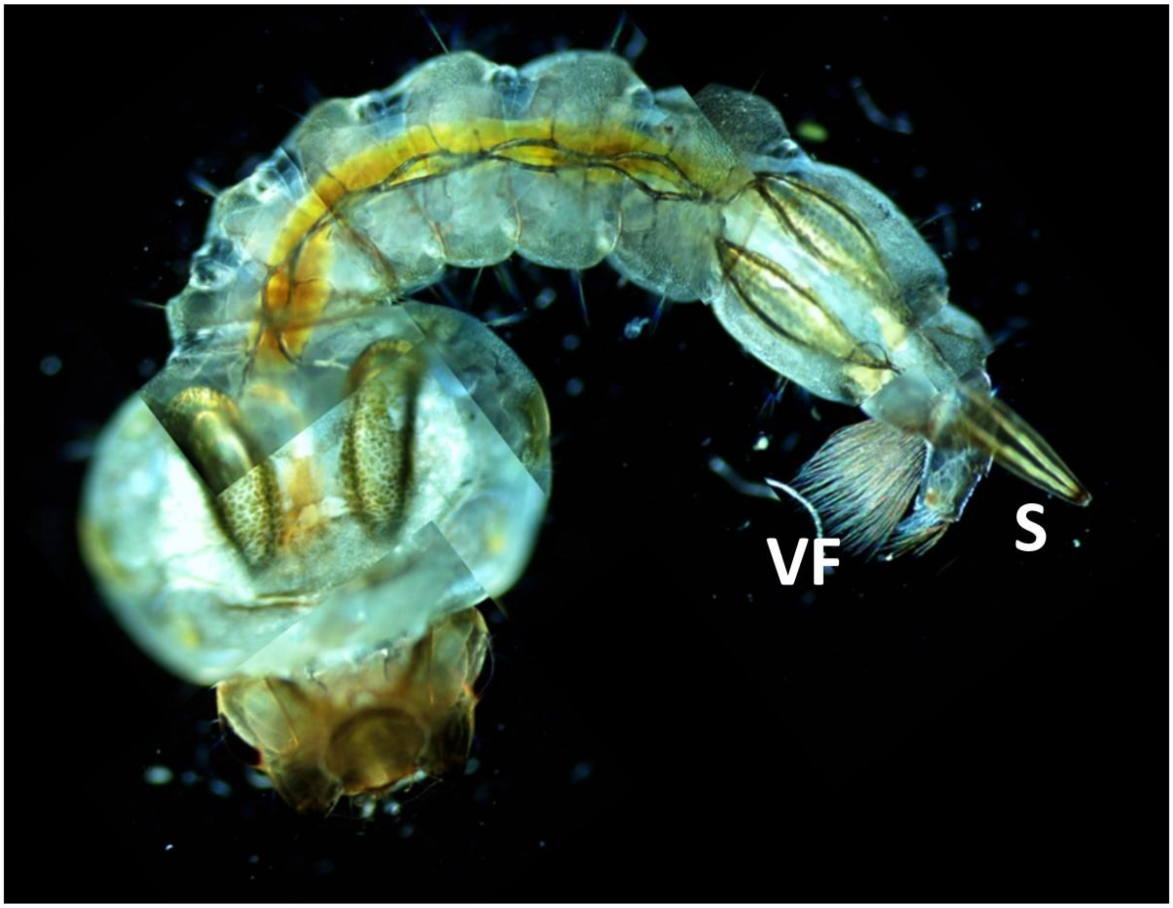

Supplemental 3. *Mochlonyx cinctipes* 4th instar larvae composite image (10x) highlighting various texture of the air bladders. Note the vestigial siphon S and the ventral fan VF. Kroph (1911) noted the trunk connecting the air bladders is liquid filled and plays no role in respiration.
